# Alteration of brain network centrality in CTN patients after a single triggering pain

**DOI:** 10.3389/fnins.2023.1109684

**Published:** 2023-02-16

**Authors:** Xiuhong Ge, Luoyu Wang, Mengze Wang, Lei Pan, Haiqi Ye, Xiaofen Zhu, Sandra Fan, Qi Feng, Quan Du, Yu Wenhua, Zhongxiang Ding

**Affiliations:** ^1^Department of Radiology Hangzhou First People’s Hospital, Zhejiang University School of Medicine, Hangzhou, Jiangsu, China; ^2^Department of Radiology, Key Laboratory of Clinical Cancer Pharmacology and Toxicology Research of Zhejiang Province, The Affiliated Hangzhou First People’s Hospital, Cancer Center, Zhejiang University School of Medicine, Hangzhou, China; ^3^Department of Radiology, The Fourth Clinical College, Zhejiang Chinese Medical University, Hangzhou, China; ^4^Department of Neurosurgery, Affiliated Hangzhou First People’s Hospital, Zhejiang University School of Medicine, Hangzhou, China

**Keywords:** classical trigeminal neuralgia, degree centrality, dynamic, function magnetic resonance imaging, central mechanism

## Abstract

**Objective:**

The central nervous system may also be involved in the pathogenesis of classical trigeminal neuralgia (CTN). The present study aimed to explore the characteristics of static degree centrality (sDC) and dynamic degree centrality (dDC) at multiple time points after a single triggering pain in CTN patients.

**Materials and methods:**

A total of 43 CTN patients underwent resting-state function magnetic resonance imaging (rs-fMRI) before triggering pain (baseline), within 5 s after triggering pain (triggering-5 s), and 30 min after triggering pain (triggering-30 min). Voxel-based degree centrality (DC) was used to assess the alteration of functional connection at different time points.

**Results:**

The sDC values of the right caudate nucleus, fusiform gyrus, middle temporal gyrus, middle frontal gyrus, and orbital part were decreased in triggering-5 s and increased in triggering-30 min. The sDC value of the bilateral superior frontal gyrus were increased in triggering-5 s and decreased in triggering-30 min. The dDC value of the right lingual gyrus was gradually increased in triggering-5 s and triggering-30 min.

**Conclusion:**

Both the sDC and dDC values were changed after triggering pain, and the brain regions were different between the two parameters, which supplemented each other. The brain regions which the sDC and dDC values were changing reflect the global brain function of CTN patients, and provides a basis for further exploration of the central mechanism of CTN.

## Introduction

According to the etiology of TN, it is classified into three subtypes, which are classical trigeminal neuralgia (CTN), secondary trigeminal neuralgia (STN) and idiopathic trigeminal neuralgia (ITN) (Olesen, 2018). CTN is a common chronic facial neurogenic disease, characterized by paroxysmal, electric shock-like pain along the trigeminal nerve branches area (Cruccu et al., 2020; Fan et al., 2022a). This pain is often triggered by non-noxious stimuli, including talking and chewing (Cruccu et al., 2020), and the pain duration often seconds to a few minutes (Shankar Kikkeri and Nagalli, 2022), while a small number of patients present sustainable pain (Di Stefano et al., 2020), and in the pain interval, often, there is no pain attack (Wang et al., 2017a). CTN is known as the most severe pain that humans can endure and often causes anxiety, depression, and other psychiatric complications (Wang et al., 2019; Bendtsen et al., 2020).

Neurovascular compression (NVC) is the main cause of CTN (Cruccu et al., 2020; Ge et al., 2022b), and its pathogenesis is caused by nerve demyelination, which leads to a short circuit between the painful and non-painful fibers, thus causing pain (Shankar Kikkeri and Nagalli, 2022). Studies on the structural and functional magnetic resonance imaging (MRI) of CTN suggested that the central nervous system is involved in the pathophysiological process of CTN (Liu et al., 2018; Wu M. et al., 2020; Wu S. et al., 2020; Zhu et al., 2020; Shankar Kikkeri and Nagalli, 2022).

In recent years, many studies have been conducted on the brain function of CTN, among which the resting state functional MRI (rs-fMRI) is a non-invasive technology applicable to several clinical diseases (Dong et al., 2021). It shows the spontaneous brain neuronal activity of the subjects and can be used as a biomarker of disease progression (Zhong and Chen, 2022). The regional homogeneity (ReHo), the amplitude of low-frequency fluctuation (ALFF), and voxel-based degree centrality (DC) are the data-driven analysis methods of rs-fMRI. ReHo reflects the temporal consistency of spontaneous neural activity between a given voxel and its neighbors (Zang et al., 2004; Ge et al., 2022a). Wang et al. (2015); Xiang et al. (2019) found that compared to healthy controls (HCs), the ReHo values of multiple brain regions are increased in CTN patients. ALFF reflects the amplitude of the given voxel time series (Firouzabadi et al., 2022). Zhang et al. (2019) demonstrated that the ALFF and fractional ALFF values were decreased in multiple brain regions in CTN patients. ReHo and ALFF reflect the local brain activity, while the DC reflects the global brain activity, which is the most direct index to measure node centrality in network analysis (Du et al., 2021).

Static DC (sDC) reflects the strength of connections between the given voxel and the other voxels in the whole brain without a prior definition of the region of interest (Bao et al., 2021). Typically, a node with a high DC value is crucial and can be regarded as the hub of information integration (Wu S. et al., 2020). The sDC has been used in TN (Zhu et al., 2020), Herpes zoster (Fan et al., 2022b), toothache (Wu S. et al., 2020), migraine (Lee et al., 2019), and other diseases. Zhu et al. (2020) showed that compared to HCs, the DC values of multiple brain regions in TN patients were significantly higher.

Although the rs-fMRI was collected in a quiet state of the subjects, the brain activity fluctuated with time (Kong et al., 2021). Dynamic DC (dDC) is a method combines sDC with “sliding window” (Tian et al., 2022), reflecting the time dynamics of remote functional connectivity (Du et al., 2021). Although dDC has not been reported in TN, it has been applied to Parkinson’s disease (Wang et al., 2022), major depressive disorder (MDD) (Yang et al., 2022), schizophrenia (Wang et al., 2021), depressive mania (Sun et al., 2022), and other diseases.

In the study, the pain was triggered by simulating the harmless actions in the daily life of CTN patients, the sDC and dDC values were measured at multiple time points after a single triggering pain. Thus, we hypothesized that: (1) the sDC and dDC values of multiple brain regions in CTN patients were altered after triggering pain; (2) the brain regions where the dDC value changes differed from that of sDC, providing a beneficial supplement to sDC.

## Materials and methods

All the participants provided written informed consent. This prospective study was approved by the local ethics committee of the Affiliated Hangzhou First People’s Hospital, Zhejiang University School of Medicine (IRB# No. 202107002). It was carried out following the Declaration of Helsinki.

### Participants

A total of 85 CTN patients were recruited from the Affiliated Hangzhou First People’s Hospital, Zhejiang University School of Medicine, between July 2021 and March 2022. The inclusion criteria for patients with CTN were as follows: (1) patients diagnosed with CTN according to the third edition of the International Classification of Headache Disorders (ICHD-3) and demonstration of NVC (not simply contact) on MRI with morphological changes (atrophy or dislocation) in the trigeminal nerve root; (2) unilateral pain in the distribution of one or more branches of the trigeminal nerve; (3) paroxysmal facial pain precipitated by trigger factors (such as light touching of the face and opening mouth); (4) conventional MRI T1 weighted imaging (T1WI) and T2WI examination showed no abnormal brain signals; (5) no additional neurological or sensory deficits in all patients; (6) no previous surgical or other invasive procedures for CTN; (7) no contraindications to MR scanning; (8) patients underwent microvascular decompression and presented NVC which not only contact; (9) right-handed patients. The exclusion criteria were as follows: (1) patients with CTN who had undergone surgical treatment before; (2) headaches and other paroxysmal or chronic pain conditions; (3) a family history of headache or other pain in first-degree relatives; (4) other somatic or psychiatric conditions; (5) left-handedness; (6) contraindications to MRI (Ge et al., 2022a).

### Experimental design

Patients on analgesic medications were asked to discontinue their medications 12 h before the scheduled scanning sessions. Before the MRI scan, a medical history was recorded to determine the zone with substantial pain in daily life. Then, the trigger zone was stimulated within 5 s before the second rs-fMRI scan; the trigger zones were stimulated by the doctors, which was a gentle touch to the patient’s trigger zone with a long cotton swab (Moisset et al., 2011). The foam was used for head fixation to ensure that the patient remained head-still during the scan. All participants underwent three-dimensional T1 weighted image (3D-T1WI) and rs-fMRI. The three time points of rs-fMRI were before stimulating the trigger zone (baseline), within 5 s after stimulating the trigger zone (triggering-5 s), and 30 min after stimulating the trigger zone (triggering-30 min). After scanning, the patients were asked whether the stimulation caused pain and whether they experienced additional pain during the scan (Ge et al., 2022b).

### Pain evaluation

If the patients experienced the stimulation, the pain would be assessed using the visual analog scale (VAS) after scanning. The researchers guided the patients in rating their pain on a scale of 0–10. A higher score indicated a greater pain intensity. A rating of “0” represented no pain, and a rating of “10” meant intolerable pain (Ge et al., 2022b).

### Image preprocessing

Resting-state function magnetic resonance imaging data preprocessing was conducted using the Data Processing and Analysis of Brain Imaging (DPABI6.1) and Statistical Parametric Mapping 12 (SPM12) toolbox based on the MATLAB platform (MathWorks, Natick, MA, USA). The preprocessing pipeline included the following steps: (1) removing the first ten time points of each session to ensure that the MRI signal reached a steady state, (2) slice-timing and head motion correction for the remaining images, (3) normalization to the Montreal Neurological Institute (MNI) and re-sampling of the resulting data to obtain 3 mm× 3 mm× 3 mm voxel size, (4) removal of the linear trend of the time course of the blood oxygenation level-dependent (BOLD) signal, and (5) a noise removal process, including the regression of Friston-24 head motion parameters, cerebrospinal fluid signals, and white matter signals. Nine patients were excluded due to large head motion (>2.5-mm maximum displacement, 2.5°rotation or framewise displacement (FD) exceeded 0.2 throughout the scanning), and the remaining 43 patients with CTN were subjected to further analysis.

### sDC calculation

For the weighted graph, DC is defined as the sum of the weights from the edges connected to the node. Compared to the binary version of DC, weighted DC provides a more accurate representation of the centrality of functional brain networks (Bao et al., 2021). Pearson’s correlation of time series between every voxel and other voxels in the whole brain calculated the correlation matrix *R* = Σ(r_*ij*_), j = 1. N-1 (R is the DC, r is the coefficient of correlation of the given voxel, j is other voxels in the whole brain, N is the number of voxels). The correlation coefficient of each voxel was *r* > 0.32 (Lv et al., 2019) (*P* < 0.05, Bonferroni correction) and were summed to obtain the weighted DC for each voxel. The threshold of 0.32 was used to eliminate the counting voxels with low temporal correlation. The different threshold choices do not alter the results qualitatively (Bao et al., 2021). For standardization, the average DC value is divided in the whole brain, and then a Gaussian kernel was used with half height and full width of 6 mm for spatial smoothing.

### dDC calculation

The sliding window method was used to calculate the time variability of the given voxel between the other voxels in the whole brain. Window length is a critical parameter in the calculation of rs-dynamics. A short window length may increase the risk of introducing spurious fluctuations in the observed dDC, and a long window length may hinder the characterization of the temporal variability dynamics of the dDC (Zang et al., 2004). In line with our previous study (Ge et al., 2022b), we used a 50 TR (100 s) sliding window length and a 2 TR (4 s) step size (Fu et al., 2021; Ge et al., 2022b; Li et al., 2022; Sun et al., 2022). Based on the method similar to sDC, after calculating the DC of all voxels in the time window, each participant will obtain multiple window-based DC graphs. Then, we calculated each participant’s standard deviation per voxel in all window-based DC plots to measure the dynamic changes in DC. In order to maintain consistency with sDC, we used a Gaussian kernel with half height and the full width of 6 mm for spatial smoothing. The step size of 5 TRs (10 s) was applied to further validate the results of dDC with different step sizes (Supplementary Figures 1, 2 and Supplementary Tables 1, 2).

### Statistical analysis

Data Processing and Analysis of Brain Imaging (DPABI) software was used to compare the sDC and dDC values of regional brain activity, measured three times for CTN patients. Repeated-measures analysis of variance (ANOVA) was used to examine the differences between the groups. The Gaussian random field theory (GRF, voxel *P* < 0.005, cluster *P* < 0.005) was applied for multiple comparison correction. Comparison between the two groups was performed using SPSS 26.0, the Bonferroni correction procedure (*P* < 0.05) was used to correct. The pearson correlation analysis was used to assess the association between the average sDC and dDC values of significant clusters and pain characteristics.

## Results

### Demographic information and clinical characteristics

**FIGURE 1:**
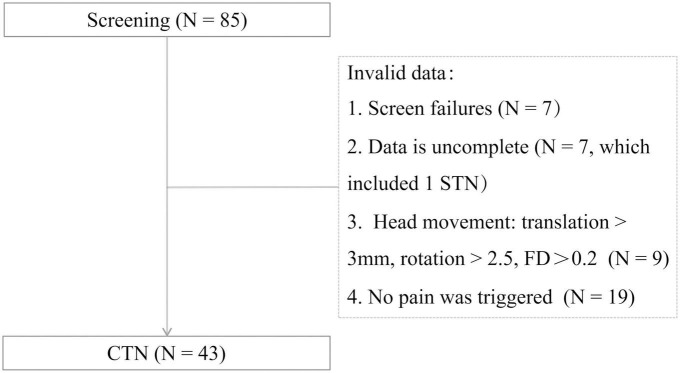
Participant selection. FD, framewise displacement; STN, secondary trigeminal neuralgia; CTN, classical trigeminal neuralgia.

A total of 43 CTN patients were included in this study. The procedures of participant selection are shown in Figure 1. The disease duration, distribution of pain, duration of each pain episode, and pain score are summarized in Table 1. And 11 patients with paroxysmal attacks lasting more than 2 min, which may be related to peripheral or central sensitization may account for the continuous pain (Olesen, 2018).

**TABLE 1:** Demographics and behavioral results of CTN.

		CTN
Men/Women		9/34
Age (years)		55.14 ± 11.59
Lateral		29R/14L
Disease duration (years)		5.14 ± 5.94
Average duration of attack (min)	<1	30
	1–2	4
	>2	9
Pain location	V2.3	25
	V3	9
	V2	7
	V1.2	2
Pain intensity (VAS)		7.69 ± 2.04

CTN, classical trigeminal neuralgia; VAS, visual analog scale.

### Compared to the baseline, the changing trend of sDC after triggering pain in CTN patients

**FIGURE 2:**
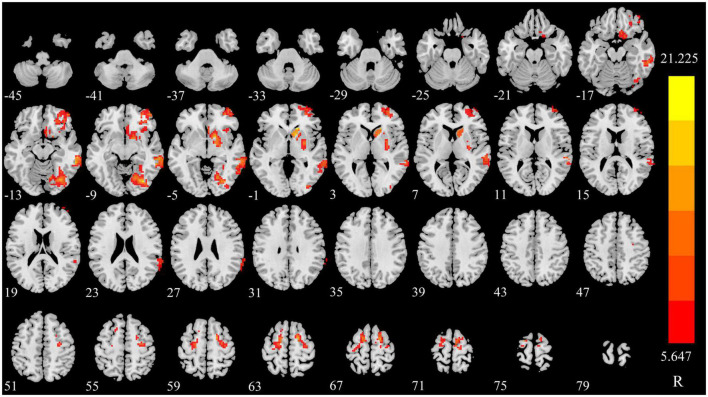
Significant differences in sDC among different time points in patients with CTN. sDC, static degree centrality; CTN, classical trigeminal neuralgia.

**FIGURE 3:**
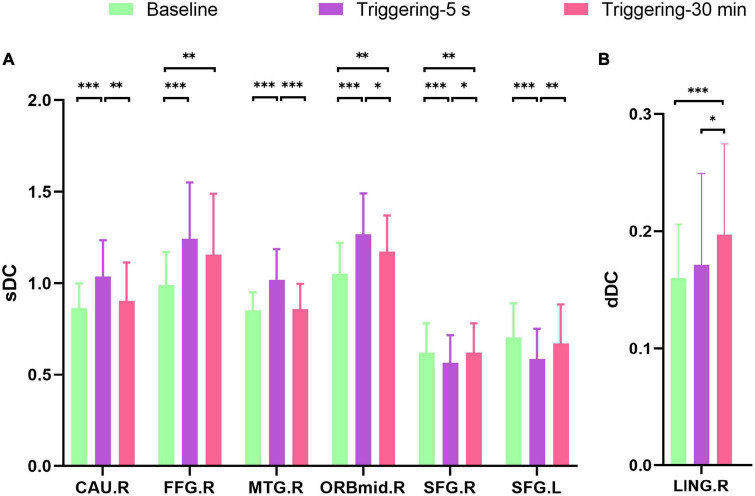
*Post hoc* comparisons of analysis of variance. The connection between two bars represents significant between-time differences of sDC **(A)** and dDC **(B)** (*represents significant level *P* < 0.05, ^**^denotes significant level *P* < 0.01, and ^***^ indicates significant level *P* < 0.001, Bonferroni correction). sDC, static degree centrality; dDC, dynamic degree centrality; baseline, the rs-fMRI was performed before stimulating the trigger zone; triggering-5 s, the rs-fMRI was performed within 5 s after stimulating the trigger zone; triggering-30 min, the rs-fMRI was performed at the 30th minute after stimulating the trigger zone. CAU.R, right caudate nucleus; FFG.R, right fusiform gyrus; MTG.R, right middle temporal gyrus; ORBmid.R, right middle frontal gyrus, orbital part; SFG.R, right superior frontal gyrus; SFG.L, left superior frontal gyrus; LING.R, right lingual.

The sDC value of right caudate nucleus (CAU), fusiform gyrus (FFG), middle temporal gyrus (MTG), middle frontal gyrus, and orbital part (ORBmid) were increased in triggering-5 s and decreased in triggering-30 min. The sDC values of bilateral superior frontal gyrus (SFG) were decreased in triggering-5 s and increased in triggering-30 min (Figures 2, 3 and Table 2). The sDC values for the three time points are listed in Supplementary Table 3.

**TABLE 2:** sDC and dDC difference in CTN patients among different timespoints.

Method	Brain region	Side	Peak MNI coordinates			Cluster size (voxels)	Peak intensity	*F*-value	*P*-value	*Post hoc P*-value		
			*X*	*Y*	*Z*					Baseline vs. 5 s	Baseline vs. 30 min	5 s vs. 30 min
sDC	CAU	R	15	24	0	305	18.934	14.041	0.000	0.000	0.164	0.000
	FFG	R	33	−75	−9	237	18.875	20.951	0.000	0.000	0.003	0.116
	MTG	R	60	−33	9	336	19.440	24.207	0.000	0.000	0.703	0.000
	ORBmid	R	45	51	−9	336	12.603	24.471	0.000	0.000	0.000	0.011
	SFG	R	15	0	63	166	14.902	24.188	0.000	0.000	0.003	0.004
	SFG	L	−15	−3	66	130	12.810	16.849	0.000	0.000	0.083	0.001
dDC	LING	R	9	−8	0	172	8.715	9.538	0.000	0.254	0.000	0.008

sDC, static degree centrality; dDC, Dynamic degree centrality; CTN, Classical trigeminal neuralgia; MNI, Montreal Neurological Institute; Baseline, the rs-fMRI was performed before stimulating the trigger zone; 5 s, the rs-fMRI was performed within 5 s after stimulating the triggerzone;30 min, the rs-fMRI was performed at the 30th min after stimulating the trigger zone; CAU, caudate nucleus; FFG, fusiform gyrus; MTG, middle temporal gyrus; ORBmid, middle frontal gyrus, orbital part; SFG, superior frontal gyrus; LING, lingual.

### Compared to the baseline, the changing trend of dDC after triggering pain in CTN patients

**FIGURE 4:**
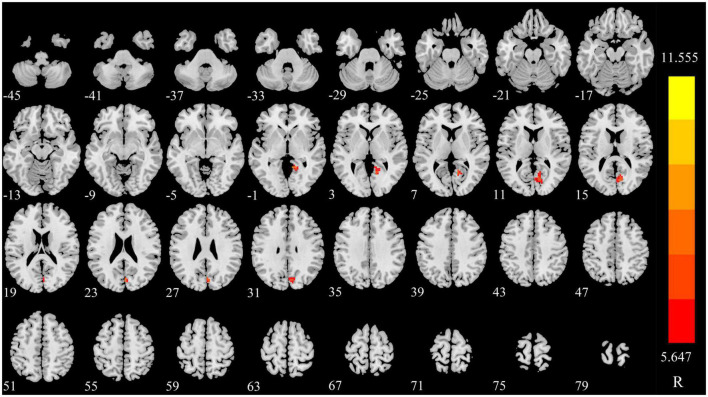
Significant differences in dDC among different time points in patients with CTN. dDC, dynamic degree centrality; CTN, classical trigeminal neuralgia.

The dDC value of right lingual (LING) was gradually increased in triggering-5 s and triggering-30 min (Figures 3, 4 and Table 2). The sDC values for the three time points are provided in Supplementary Table 3.

### The correlation between the average sDC and dDC values of significant brain regions and the pain characteristics

**FIGURE 5:**
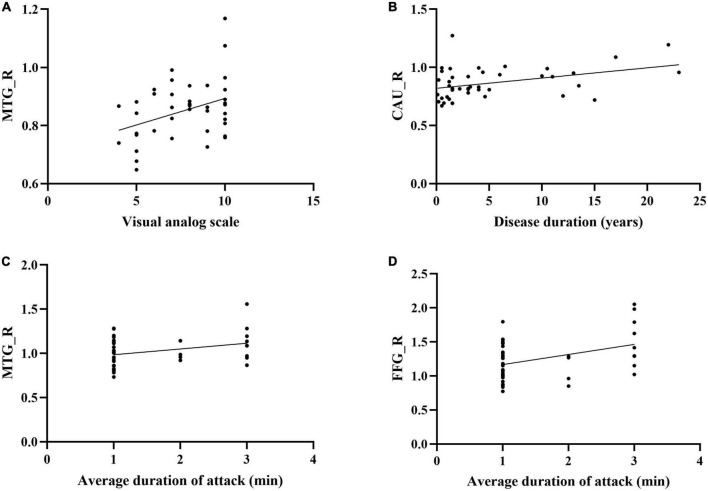
Correlations between the clinical parameters and brain regions which the sDC were changed in CTN patients. **(A)** The sDC value of MTG_R was positively correlated with the visual analog scale (*P* = 0.013, *r* = 0.377). **(B)** The sDC value of CAU_R was positively correlated with the disease duration (years) (*P* = 0.010, *r* = 0.390). **(C)** The sDC value of MTG_R was positively correlated with the average duration of attack (*P* = 0.036, *r* = 0.321). **(D)** The sDC value of FFG_R was positively correlated with the average duration of attack (*P* = 0.008, *r* = 0.399). sDC, static degree centrality; CTN, Classical trigeminal neuralgia; Baseline, the rs-fMRI was performed before stimulating the trigger zone; triggering-5 s, the rs-fMRI was performed within 5 s after stimulating the trigger zone; CAU_R, right caudate nucleus; FFG_R, right fusiform gyrus; MTG_R, right middle temporal gyrus.

The sDC value of CAU in baseline was positively correlated with the disease duration. The sDC value of MTG in baseline was positively correlated with VAS. The sDC values of FFG in baseline and triggering-5 s were both positively associated with pain persistence (Figure 5).

## Discussion

In the previous study, we explored the local brain function after a single triggering pain in CTN patients, namely, the time-frequency properties of ALFF (static ALFF, dynamic ALFF) (Ge et al., 2022b). In this study, we analyzed the changes in global brain function at different times after a single triggering pain and found that (1) the sDC and dDC values of multiple brain regions were changed after triggering pain; (2) the brain regions which the dDC value altered are different from those of the sDC, which is complementary to sDC and provides a new perspective and supplement for exploring the central mechanism of CTN patients.

CTN is known as the most severe pain that humans can endure. Currently, most studies on the brain function of CTN patients are based on the cross-sectional rs-fMRI (Xiang et al., 2019; Wu M. et al., 2020; Zhu et al., 2020; Li et al., 2021; Zhang et al., 2021; Liu et al., 2022). Several studies are based on the task state of TN with fewer subjects, including one case report which studied the local brain function (Borsook et al., 2007). DC is one of the methods to evaluate the changes in brain functional network activity, which can detect the functional importance of different nodes in the brain at the voxel level (Wu S. et al., 2020). Although DC has been applied to various diseases (Keefe et al., 1991; Zang et al., 2004; Xu et al., 2019; Li et al., 2020; Serafini et al., 2020; Wu S. et al., 2020; You et al., 2020; Dong et al., 2021; Ma et al., 2021; Zhong and Chen, 2022), there has been less research on TN. In this study, we analyzed the changing trend of sDC and dDC at multiple time points after triggering pain in CTN patients. It not only simulates the changing trend of the whole brain functional connectivity after triggering pain in CTN patients and provides a basis for exploring its central mechanism but also avoids discomfort or uncooperating caused by the pain task states.

In this study, the sDC values of the right CAU, MTG, FFG, and ORBmid were changed in the same trend, i.e., increased in triggering-5 s and decreased in triggering-30 min, indicating that the connectivity with the whole brain increased significantly for a short period after triggering pain, and then recovered gradually in the four brain regions. However, the recovery levels in triggering-30 min were slightly different, indicating that the four regions participate in the pain process of CTN, but the specific role is diverse.

The CAU is a key region of the subcortical network that receives damaging information from the trigeminal nucleus through direct projection from the trigeminal spinal cord nucleus. In addition, the CAU plays a crucial role in assessing the consistency between actions and outcomes (Zhang et al., 2021). The MTG is a major part of the default mode network (DMN) and is also involved in the attention network (Yeo et al., 2011). The changing trend of sDC in the right MTG indicates its role in the pain process but not as a component of DMN, but may be as a part of the attention network. Additionally, CTN patients with long-term pain may sensitize the resting state and further increase their connectivity after triggering pain. However, the specific mechanism needs to be studied further. The FFG is located on the basal surface of the temporal and occipital lobes and is involved in various sensory integration and cognitive processing. It is also an integral part of the limbic system that is closely associated with mental abilities, such as emotion, behavior, learning, and memory. FFG plays a crucial role in the anticipation and perception of pain regulation (Wang et al., 2017a). Li et al. (2017) showed that compared to HCs, the gray matter volume of bilateral MTG, CAU, and right FFG was reduced in CTN patients. Zhang et al. (2020) conducted a meta-analysis of brain function changes in CTN patients and found that the signal in MTG was inconsistent across studies. This phenomenon could be attributed to paroxysmal and transient CTN pain. Different studies put forth varied results in terms of whether the patients have pain and different frequencies of pain.

The SFG located in the upper part of the prefrontal cortex is involved in socially oriented thoughts and may also be involved in anticipation of impending pain (Torrado Pacheco et al., 2021). The SFG is also involved in the composition of DMN, which is active in the resting state (Yuan et al., 2018; Zhang et al., 2019), and the activity is reduced when involved in tasks and stimuli (including painful stimuli) (Wang et al., 2017b). This finding is consistent with the changing trend of bilateral SFG in this study, i.e., the sDC value was decreased in triggering-5 s and increased in triggering-30 min. This indicates that the functional importance of the node decreases for a short period after the triggering pain and recovers gradually. Yuan et al. (2018) found that compared to HCs, the ReHo of the SFG in idiopathic TN patients was significantly increased. Xiang et al. (2019) showed that the ReHo value of right SFG in CTN patients was increased compared to that in HCs. SFG may be a leading node in the brain network that coordinates working memory (Alagapan et al., 2019). The pain of CTN patients is triggered by harmless movements in daily life, and the patients may deliberately restrict such movements (for example, chewing and speaking) to avoid pain. Therefore, we speculated that the signal changing of the SFG is not only related to pain but also related to the memory formed by the patients’ daily movement restriction.

The dDC reflects the time variability of DC, i.e., the fluctuation of DC as time changes. In this study, after triggering pain in CTN patients, we found a brain region with obviously changed dDC value, namely, the right LING, and was different from the brain regions with altered sDC value, indicating that dDC provides additional Supplementary Information. The LING is a part of the visual processing network (Russo et al., 2019). The dDC value of the right LING was decreased in triggering-5 s and increased in triggering-30 min, indicating that the variability of dDC value of the LING was decreased in a short period and then increased. Zhu et al. (2020) found that compared to HCs, the DC value of the right LING in TN patients was significantly higher.

## Limitations

In this study, we explored the changing trend of static and dynamic functional connections in CTN patients at multiple time points after triggering pain. Compared to the baseline, the sDC or dDC values of each brain region showed a recovery trend at triggering-30 min, but the recovery level was different and did not return to the baseline, which required a prolonged duration to further clarify the changing trend in the brain functional connectivity after triggering pain. Secondly, we did not conduct the classification studies according to the average duration of attack, and in the future, we will study CTN patients depending on the average duration of attack.

## Conclusion

After a single triggering pain, both the sDC and dDC values of CTN patients were changed in different brain regions, which were complementary to each other. These brain regions which the sDC and dDC values were changing reflected the global brain function of CTN patients and provided a specific basis for further exploration of the central mechanism of CTN.

## Data availability statement

The original contributions presented in this study are included in this article/Supplementary material, further inquiries can be directed to the corresponding author.

## Ethics statement

Written informed consent was obtained from the individual(s) for the publication of any potentially identifiable images or data included in this article.

## Author contributions

ZD, HY, and QD: study concept or design and revisions to the manuscript. XG: data acquisition and analysis and drafting and writing of the manuscript. LW: data analysis and revisions to the manuscript. MW: make important revisions to the manuscript and edit language. LP, HY, QF, and XZ: data collection. SF: language editing and revisions to the manuscript. All authors contributed to the article and approved the submitted version.
