# Supplementary material for: Alteration of brain network centrality in CTN patients after a single triggering pain
Source: Front Neurosci. 2023 Feb 16;17:1109684. doi: 10.3389/fnins.2023.1109684 (PMC9978223; doi:10.3389/fnins.2023.1109684)
Supplement: Supplementary file 1 [file Data_Sheet_1.pdf]

Table S1 The dynamic degree centrality difference in CTN patients among different time points with the step size of 5 TRs (10 s).

Brain region	Side	Peak MNI coordinates			Cluster size (voxels)	Peak intensity	*F* value	*P* value	*Post hoc P* value		
		X	Y	Z					Baseline vs 5 s	Baseline vs 30 min	5 s vs 30 min
LING	R	9	-48	0	154	8.547	9.498	0.000	0.750	0.000	0.025

MNI, Montreal Neurological Institute; Baseline, the rs-fMRI was performed before stimulating the trigger zone; 5 s, the rs-fMRI was performed within 5 s after stimulating the trigger zone; 30 min, the rs-fMRI was performed in the 30th minute after stimulating the trigger zone; LING.R, right lingual; right; l; R

Table S2. The dynamic degree centrality value (step size of 5 TRs (10 s)) in CTN patients in different time points.

Brain region	Side	dDC value		
		Baseline	5s	30min
LING	R	0.161 ± 0.046	0.173± 0.079	0.199 ± 0.078

CTN , classical trigeminal neuralgia; dDC,dynamic degree centrality; Baseline, the rs-fMRI was performed before stimulating the trigger zone; 5 s, the rs-fMRI was performed within 5 s after stimulating the trigger zone; 30 min, the rs-fMRI was performed in the 30th minute after stimulating the trigger zone; LING, lingual; R, right.

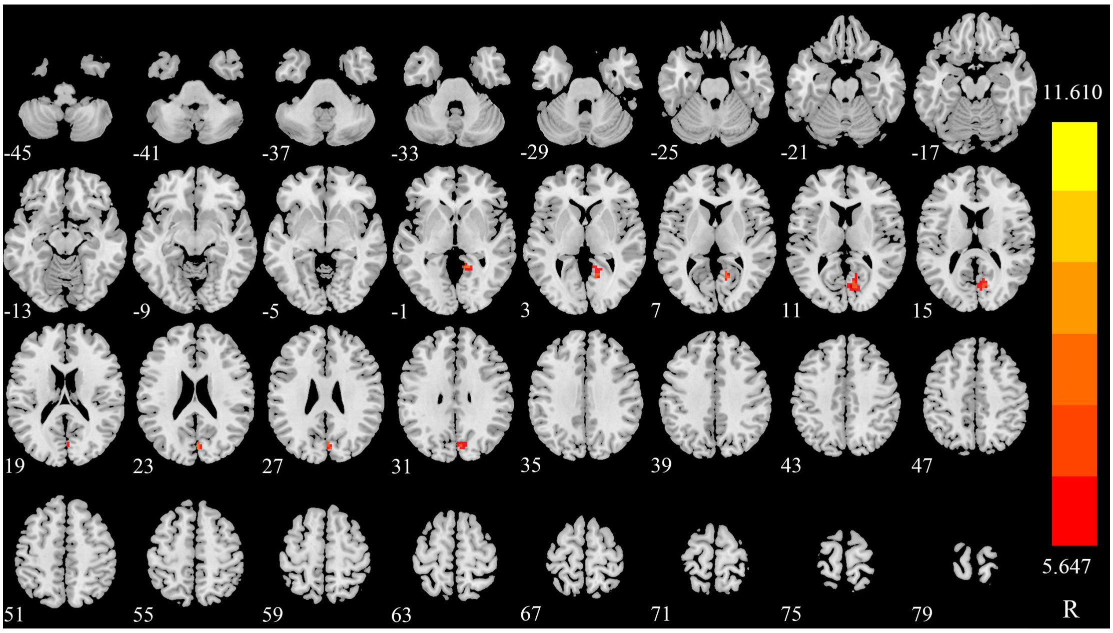

Figure S1. Significant differences in dDC among different times in patients with CTN. *dDC, Dynamic degree centrality; CTN, classical trigeminal neuralgia.*

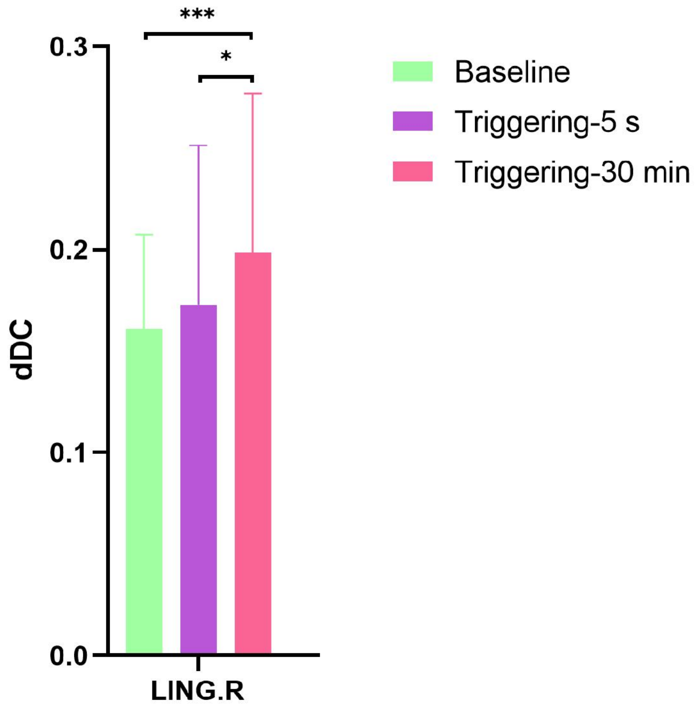

Figure S2. *Post hoc* comparisons of analysis of variance. *The connection between two bars represents significant between-time differences of dDC (*represents significant level P < 0.05, **denotes significant level P < 0.01, and *** indicates significant level P < 0.001, Bonferroni correction). dDC, Dynamic degree centrality; baseline, the rs-fMRI was performed before stimulating the trigger zone; triggering-5 s, the rs-fMRI was performed within 5 s after stimulating the trigger zone; triggering-30 min, the rs-fMRI was performed in the 30th minute after stimulating the trigger zone; LING.R, right lingual.*

Table S3 The sDC and dDC values in CTN patients among different time points.

Method	Brain region	Side	value		
			Baseline	5s	30min
sDC	CAU	R	0.865±0.134	1.036±0.199	0.904±0.208
	FFG	R	0.991±0.179	1.243±0.307	1.156±0.332
	MTG	R	0.851±0.099	1.018±0.168	0.858±0.138
	ORBmid	R	1.050±0.169	1.267±0.223	1.171±0.199
	SFG	R	0.674±0.148	0.566±0.151	0.621±0.161
	SFG	L	0.703±0.187	0.585±0.167	0.670±0.214
dDC*	LING	R	0.160±0.046	0.171±0.078	0.197±0.077

sDC, static degree centrality; dDC, Dynamic degree centrality; Baseline, the rs-fMRI was performed before stimulating the trigger zone; 5 s, the rs-fMRI was performed within 5 s after stimulating the trigger zone; 30 min, the rs-fMRI was performed in the 30th minute after stimulating the trigger zone; sDC, static degree centrality; dDC, dynamic degree centrality; CAU, caudate nucleus; FFG, fusiform gyrus; MTG, middle temporal gyrus; ORBmid, middle frontal gyrus, orbital part; SFG, superior frontal gyrus; LING, lingual; R, right; L, left. *step size of 2 TRs (4 s) of dDC in different time.
